# Latent Profiles of Post-Stroke Fatigue and Their Association with Anxiety, Depression, and Sleep in Patients with First-Ever Stroke: A Cross-Sectional Study

**DOI:** 10.3390/healthcare14152383

**Published:** 2026-08-04

**Authors:** Jiajia Lai, Yuyan Yang, Xiaohui Liu, Jialin Yuan, Shailing Ma, Yingjie Zheng, Yijia Qi, Jing Li

**Affiliations:** 1 School of Nursing, Ningxia Medical University, Yinchuan 750004, China; laijiajia@nxmu.edu.cn (J.L.); mashailing@nxmu.edu.cn (S.M.); zhengyingjie@nxmu.edu.cn (Y.Z.); qiyijia1005@nxmu.edu.cn (Y.Q.); ljing@nxmu.edu.cn (J.L.); 2 General Hospital of Ningxia Medical University, Yinchuan 750001, China; yuyan3y@hotmail.com; 3 The First People’s Hospital of Yinchuan, Yinchuan 750001, China; 18709543351@163.com

**Keywords:** stroke, post-stroke fatigue, sleep, anxiety symptoms, depressive symptoms, latent profile analysis

## Abstract

**Highlights:**

**What are the main findings?**

- Three distinct latent profiles of post-stroke fatigue were identified among first-ever stroke patients: low-, moderate-, and high-fatigue subgroups.
- Patients in higher fatigue profiles experienced more severe anxiety symptoms, depressive symptoms, and sleep disturbances, with a graded association between fatigue severity and psychological-sleep disturbance.

**What are the implications of the main findings?**

- Screening stroke survivors for fatigue subtypes may help clinicians identify high-risk groups with concurrent emotional and sleep disturbances, facilitating targeted early intervention.
- The classification framework provides a foundation for personalized stroke rehabilitation and symptom management research.

**Abstract:**

**Objectives:** This study aimed to identify latent profiles of post-stroke fatigue (PSF) and examined their associations with anxiety symptoms, depressive symptoms, and sleep quality among first-ever stroke survivors. **Methods:** From December 2023 to May 2024, participants were recruited via convenience sampling from the Neurology Department of a tertiary hospital in Yinchuan, Ningxia. Data were collected using the Fatigue Severity Scale (FSS), the Athens Insomnia Scale (AIS), and the Hospital Anxiety and Depression Scale (HADS). Latent profile analysis (LPA) was performed using Mplus 8.3. Intergroup differences in psychological symptoms and sleep quality were compared using the Bolck–Croon–Hagenaars (BCH) method. **Results:** Three distinct latent profiles were identified: low FSS profile (28.0%), subthreshold intermediate FSS profile (30.2%), and high FSS profile (41.8%). Compared with the low FSS profile, the subthreshold intermediate FSS profile reported significantly higher levels of anxiety symptoms, depressive symptoms, and sleep disturbances. Furthermore, the high FSS profile exhibited the most severe symptoms across all three variables compared to both other groups (*p* < 0.05). Only the high FSS profile exceeded the clinical cut-off for fatigue (FSS ≥ 4). **Conclusions:** Post-stroke fatigue exhibits substantial heterogeneity among first-ever stroke patients and is closely linked to psychological distress and poor sleep quality. Clinical interventions should target emotional distress and sleep disorders simultaneously through individualized rehabilitation to potentially enhance patient recovery and quality of life.

## 1. Introduction

Stroke is a primary driver of long-term disability and the second leading cause of death worldwide [1,2]. The prevalence of stroke has been rising in China [3]. Recent estimates indicate that approximately 17.8 million people were living with stroke in China in 2020, with about 3.4 million new cases and 2.3 million deaths annually [4]. These cerebrovascular events often lead to persistent physical disabilities and are frequently accompanied by cognitive, psychological, and behavioral impairments [5,6]. Consequently, such sequelae considerably reduce the health-related quality of life (HRQoL) among survivors [7]. Beyond individual morbidity, the condition also imposes a heavy socioeconomic burden and places considerable strain on families and the healthcare system [8].

### Background

Post-stroke fatigue (PSF) is clinically defined as an overwhelming sense of lassitude that arises independently of physical exertion and is largely refractory to rest [9]. Emerging evidence suggests that the prevalence of PSF-related symptoms exceeds 50% among stroke survivors worldwide [10]. Notably, approximately 40% of patients identify fatigue as the most debilitating post-stroke sequela [11]. Recognized as a common disabling condition following stroke [12], PSF can manifest at any phase of the disease trajectory and often persists chronically [13]. PSF is associated with poorer rehabilitation outcomes and significant constraints on activities of daily living (ADL) and occupational functioning [14]. Furthermore, PSF is negatively associated with patients’ subjective appraisal of functional recovery [15], with reduced adherence to exercise-based rehabilitation, and with an elevated risk of suicidal ideation. Recent longitudinal studies suggest that individuals with PSF have higher three-year mortality rates following stroke onset compared to those without fatigue [16,17]. Thus, precise and targeted interventions are urgently required to address the development and long-term impact of PSF.

Stroke is associated with adverse psychological sequelae, including anxiety and depressive symptoms, which are related to protracted treatment courses, extensive physical deficits, and poor prognoses. Evidence suggests that anxiety, depressive symptoms, and sleep disorders are associated with the severity of PSF [18]. In clinical practice, PSF is occasionally misattributed to a secondary manifestation of anxious-depressive disorders, thereby overlooking its distinct pathophysiological characteristics. This diagnostic bias may stem from the traditional conceptualization of fatigue as a core symptomatic criterion for anxiety and depression. As a result, previous research has often conflated post-stroke anxiety, depressive symptoms, and fatigue into a single clinical construct. However, emerging data indicate that a subset of patients experiences fatigue in the absence of concurrent affective symptoms. Furthermore, research has shown that while antidepressant and anxiolytic pharmacotherapy significantly reduces depressive scores, fatigue symptoms often persist, suggesting a distinct etiology [19,20].

Sleep disorders are common post-stroke complications that are closely associated with the development of fatigue. A longitudinal cohort study [21] reported that three years post-stroke, 81% of patients experienced sleep-disordered breathing, while 20% had sleep apnea syndrome—symptoms that frequently overlap with fatigue. Furthermore, research by Zeng et al. [22] showed that patients who experienced sleep disorders during the acute phase of stroke were more likely to develop fatigue compared to those without sleep disorders. This association may be partly explained by impaired sleep quality and its negative impact on cognitive function. Additionally, chronic sleep disruption may influence physiological homeostasis and is associated with an increased risk and severity of fatigue.

A significant limitation in the existing literature is the failure to account for inter-individual heterogeneity among patients with PSF. Specifically, patients manifest varying degrees of symptom severity that cannot be adequately explained through simple univariate associations. Latent profile analysis (LPA) can identify relationships among observed continuous indicators through latent class variables, allowing the associations among these observed indicators to be estimated while maintaining local independence [23]. By objectively identifying distinct phenotypic subgroups, LPA provides a robust framework for evidence-based clinical decision-making, which can help identify high-risk populations and develop targeted interventions. For instance, Zhou et al. [24] utilized LPA to analyze social participation among stroke survivors, identifying four distinct subgroups shaped by physical function, psychological resilience, and sociodemographic factors, which may help guide individualized social participation intervention strategies. Similarly, research by Liu et al. [25] highlighted the phenotypic diversity of “fear of disease progression,” suggesting that tailored psychological support strategies are essential. Collectively, these studies show the substantial utility of LPA in advancing stroke-related clinical research.

Despite these contributions, certain gaps remain. First, most existing person-centered studies on post-stroke fatigue have focused on longitudinal trajectories, whereas cross-sectional profiling of fatigue subtypes in first-ever stroke patients—a critical window for early identification and intervention—remains relatively underexplored. Second, although the interconnections among fatigue, anxiety, depressive symptoms, and sleep disturbances are well recognized, few studies have simultaneously examined the associations among these three dimensions within a single analytical framework. Third, person-centered studies of post-stroke fatigue focusing specifically on first-ever stroke patients in the Chinese population are still limited. Therefore, the present study aims to address these gaps by: (a) applying latent profile analysis to identify distinct subtypes of post-stroke fatigue in Chinese first-ever stroke patients; (b) examining the associations between these fatigue subtypes and anxiety, depressive symptoms, and sleep quality; (c) providing empirical evidence that may inform early screening and targeted interventions for high-risk patients.

This cross-sectional study aims to identify latent profiles of PSF in patients with first-ever stroke through latent profile analysis and to examine differences in anxiety, depressive symptoms, and sleep among the resulting subgroups. The aim is to delineate the factors associated with distinct PSF types, with the aim of characterizing the heterogeneity of PSF in this population and providing novel scientific evidence that may inform targeted intervention strategies and improve patients‘ quality of life.

## 2. Methods

A convenience sampling approach was employed to recruit patients presenting with their first-ever stroke event who were admitted to the Department of Neurology at a tertiary Grade A hospital in Yinchuan, Ningxia Province, China, between December 2023 and May 2024.

The inclusion criteria were as follows: ① Diagnostic criteria: patients diagnosed with stroke were those who met the diagnostic criteria set forth in the Chinese Guidelines for Diagnosis and Treatment of Intracerebral Haemorrhage (2019) [26] and the Chinese Guidelines for Diagnosis and Treatment of Acute Ischaemic Stroke (2018) [27], with diagnosis confirmed by CT or MRI imaging, presenting with a first-ever confirmed diagnosis and an onset time within 2 weeks (i.e., within the acute phase); ② age ≥ 18 years; ③ clear consciousness and sufficient communicative capacity to engage with study protocols; ④ voluntary provision of written informed consent.

Patients were excluded based on the following criteria: ① with severe paralysis (including upper extremity and lower extremity); ② recent exposure to significant life stressors; ③ a documented history of depressive disorders within three months prior to stroke onset; ④ cancer patients; ⑤ severe cardiac, hepatic, or renal insufficiency, or systemic infection; ⑥ attrition or withdrawal during the study period.

Following the statistical guideline of 10 to 20 observations per independent variable, a minimum sample size was calculated based on the nine identified independent variables. After adjusting for a predicted 20% attrition rate, the required sample size was estimated at 108 to 216 participants.

### 2.1. Survey Tools

Data collection was conducted through the concurrent administration of a comprehensive survey battery, comprising a study-specific demographic questionnaire and three validated psychometric instruments: the Fatigue Severity Scale (FSS), the Hospital Anxiety and Depression Scale (HADS), and the Athens Insomnia Scale (AIS). These standardized scales enabled a multidimensional assessment of the participants’ physiological and psychological states.

#### 2.1.1. Demographic Questionnaire

The demographic questionnaire consisted of two primary sections: sociodemographic characteristics and clinical data. Sociodemographic variables included age, sex, residence, educational attainment, marital status, employment status, monthly income, and the presence of comorbidities. Clinical parameters focused on stroke-specific factors, including the location and number of lesions, the total number of comorbid chronic diseases, and National Institutes of Health Stroke Scale (NIHSS) scores.

#### 2.1.2. Fatigue Severity Scale (FSS)

The scale was translated into Chinese by Wu Chunwei et al. [28] in 2007 and validated in stroke patients, yielding a Cronbach‘s α of 0.929, which indicates good internal consistency. The scale comprises 9 items rated on a 7-point Likert scale ranging from 1 (strongly disagree) to 7 (strongly agree). The total FSS score is calculated as the mean of all item scores, with FSS ≥ 4 indicating fatigue and FSS < 4 indicating no fatigue. In the present study, the scale demonstrated good reliability and validity.

#### 2.1.3. Hospital Anxiety and Depression Scale (HADS)

Originally developed by Zigmond and Snaith in 1983 [29], the Chinese version of the Hospital Anxiety and Depression Scale (HADS) is a 14-item self-assessment instrument, comprising two subscales, namely the Anxiety subscale and the Depression subscale. The total score of the Chinese version of HADS ranges from 0 to 42, whereas each subscale yields a score ranging from 0 to 21. A score exceeding 7 on either the Anxiety subscale or the Depression subscale is indicative of the presence of anxiety or depressive symptoms, respectively; furthermore, a score of 8 or higher on both subscales is considered to signify “comorbid anxiety and depressive symptoms.” The Cronbach‘s α coefficients for the overall Chinese version of HADS, the Anxiety subscale, and the Depression subscale are 0.879, 0.806, and 0.806, respectively.

#### 2.1.4. Athens Insomnia Scale (AIS)

This scale was introduced into Chinese through the adaptation by Chiang et al. [30] in 2009 and serves as a self-assessment instrument for evaluating sleep disturbances in individuals. The scale consists of a total of 8 items, all of which are rated using a Likert 4-point scoring method, where responses ranging from “none” to “severe” are assigned scores of 0 to 3, respectively. The total score ranges from 0 to 24, with higher scores indicating poorer sleep quality; moreover, a score exceeding 6 suggests the presence of sleep disorders in the patient. In the present study, the Cronbach‘s α coefficient for this scale was 0.825, indicating satisfactory reliability and validity.

### 2.2. Data Collection and Quality Control

Participants were recruited in strict accordance with predefined inclusion and exclusion criteria. Prior to administration, researchers briefed participants on the study objectives and protocols, and written informed consent was obtained. For participants unable to complete the questionnaire independently, researchers provided standardized, non-directive assistance to ensure comprehension. Questionnaires were administered and collected on-site; any omissions or ambiguities were addressed immediately to ensure data integrity. Of the 596 distributed questionnaires, 46 were excluded prior to final analysis. Among them, 16 were incomplete or invalid, while 30 were excluded due to limited participant cooperation or inadequate data quality. Ultimately, 550 valid questionnaires were retained, yielding an effective response rate of 92.28%.

### 2.3. Statistical Analysis

Data analysis was performed using SPSS version 27.0. Normally distributed continuous variables were reported as mean ± standard deviation ($\bar{x}$ ± s), while non-normally distributed variables were presented as medians with interquartile ranges (IQR). Categorical and ordinal variables were described using frequencies and percentages. For group comparisons, categorical variables were compared using the chi-square (χ^2^) test, and non-normally distributed continuous variables were compared using the Kruskal–Wallis H test. Statistical significance was set at *p* < 0.05. LPA of fatigue severity was performed using Mplus version 8.3, where the following indices were employed: the Akaike Information Criterion (AIC), the Bayesian Information Criterion (BIC), the sample-size adjusted BIC (aBIC), and entropy, along with model comparison indicators including the Lo-Mendell–Rubin adjusted likelihood ratio test (LMR) and the Bootstrap likelihood ratio test (BLRT). Lower AIC, BIC, and aBIC values indicate better fit. When both the LMR and BLRT are significant (*p* < 0.05), a k-class model fits better than a (k-1)-class model. Entropy ranges from 0 to 1, with values > 0.80 indicating good classification accuracy. It is also recommended that the smallest class comprise at least 5% of the total sample [31]. The Bolck–Croon–Hagenaars (BCH) method, a procedure for testing mean differences in continuous outcomes across latent profile groups [32], has been recommended for the evaluation of auxiliary variables in LPA [33]. After subgroup classification, we used the BCH method to compare three auxiliary outcome variables—anxiety symptoms, depressive symptoms, and sleep quality—across the three latent fatigue profiles. No covariates were adjusted in these comparisons, as the BCH method is designed to test auxiliary variable differences while accounting for classification error and preserving the latent class structure.

### 2.4. Ethical Considerations

This study protocol was approved by the Ethics Committee of Ningxia Medical University (Approval No. NXMU Ethics 2023-056). All participants provided written informed consent prior to enrollment. 1. Communicate and explain well with the relevant departments and heads of departments in the hospital to obtain permission and support for this study. 2. Explain the purpose and significance of the study as well as the general process of the study to the study participants, obtain the support and cooperation of the patients, and promise to protect the patients‘ private information and never disclose any information about the patients’ illnesses throughout the study. 3. Patients voluntarily participate in the study and sign an informed consent form.

## 3. Results

### 3.1. General Demographic Data of First-Ever Stroke Patients

The study enrolled 550 patients with first-ever stroke, of whom 377 were male (68.5%) and 173 were female (31.5%). In terms of age distribution, 51 patients (9.3%) were aged 18–45 years, 193 (35.1%) were aged 46–60 years, and 306 (55.6%) were aged >60 years. Regarding educational attainment, 244 patients (44.4%) had completed primary education or below, 157 (28.5%) had finished junior secondary school, 85 (15.5%) had completed senior secondary or vocational school, and 64 (11.6%) had attained a bachelor’s degree or higher. Detailed demographic characteristics are provided in Table 1.

### 3.2. Latent Profile Analysis of Fatigue in First-Ever Stroke Patients

Models from one to five classes were evaluated (Table 2). As the number of classes increased, AIC, BIC, and aBIC values decreased, with the lowest values in the five-class model. The three- and four-class models both had entropy > 0.8, indicating good classification accuracy. The LMR and BLRT were significant (*p* < 0.05) for both. Model selection thus balanced statistical fit, parsimony, clinical interpretability, and theoretical coherence.

The three-class model was chosen over the four-class model for several reasons. First, the smallest class in the four-class solution represented only 11.1% of the sample—above the 5% rule of thumb, but sufficiently small to raise concerns about parameter stability. Second, posterior probabilities for the three-class model were high (0.954–0.995), whereas the smallest class in the four-class solution had a lower probability (0.915), suggesting weaker classification. Third, the three classes were readily interpretable in clinical terms—low FSS profile, subthreshold intermediate FSS profile, and high FSS profile—and each showed a distinct, internally consistent pattern of item responses.

In summary, the three-class model was selected for its balance of parsimony, interpretability, and classification quality. The three profiles—low FSS profile (28.0%), subthreshold intermediate FSS profile (30.2%), and high FSS profile (41.8%)—offer a clinically useful framework for understanding fatigue heterogeneity and guiding targeted interventions in first-ever stroke patients. Only the high FSS profile exceeded the clinical FSS cut-off of 4.

**Figure 1:**
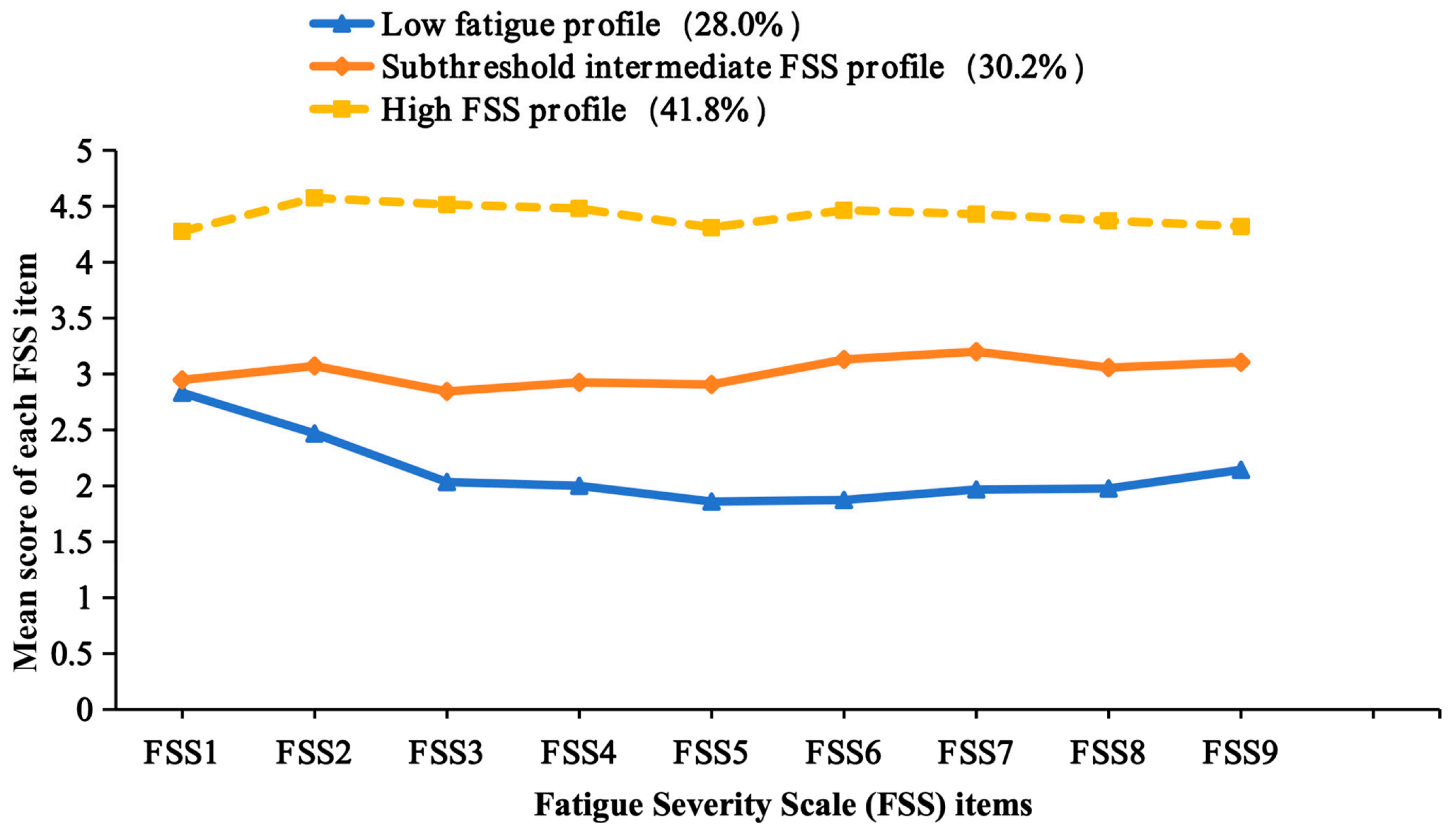
Mean item scores of the three post-stroke fatigue latent profiles based on the Fatigue Severity Scale (FSS).

Class 1 (n = 154, 28.0%) had uniformly low scores across all nine FSS items, with an overall mean of 2.13 (95% CI: 2.01–2.25)—well below the clinical cut-off of 4. We designated this class the “low FSS profile.” Class 2 (n = 166, 30.2%) showed intermediate scores, with a mean of 3.02 (95% CI: 2.90–3.14), still below the FSS threshold of 4. To avoid misinterpretation, we labeled this class the “subthreshold intermediate FSS profile”—elevated relative to Class 1, but not reaching clinical fatigue. Class 3 (n = 230, 41.8%) had the highest scores across all items, with a mean of 4.42 (95% CI: 4.37–4.47), exceeding the clinical FSS cut-off of 4. We classified this group as the “high FSS profile” (Figure 1).

### 3.3. Association of Fatigue with Anxiety, Depression, and Sleep Among First-Ever Stroke Patients

**Figure 2:**
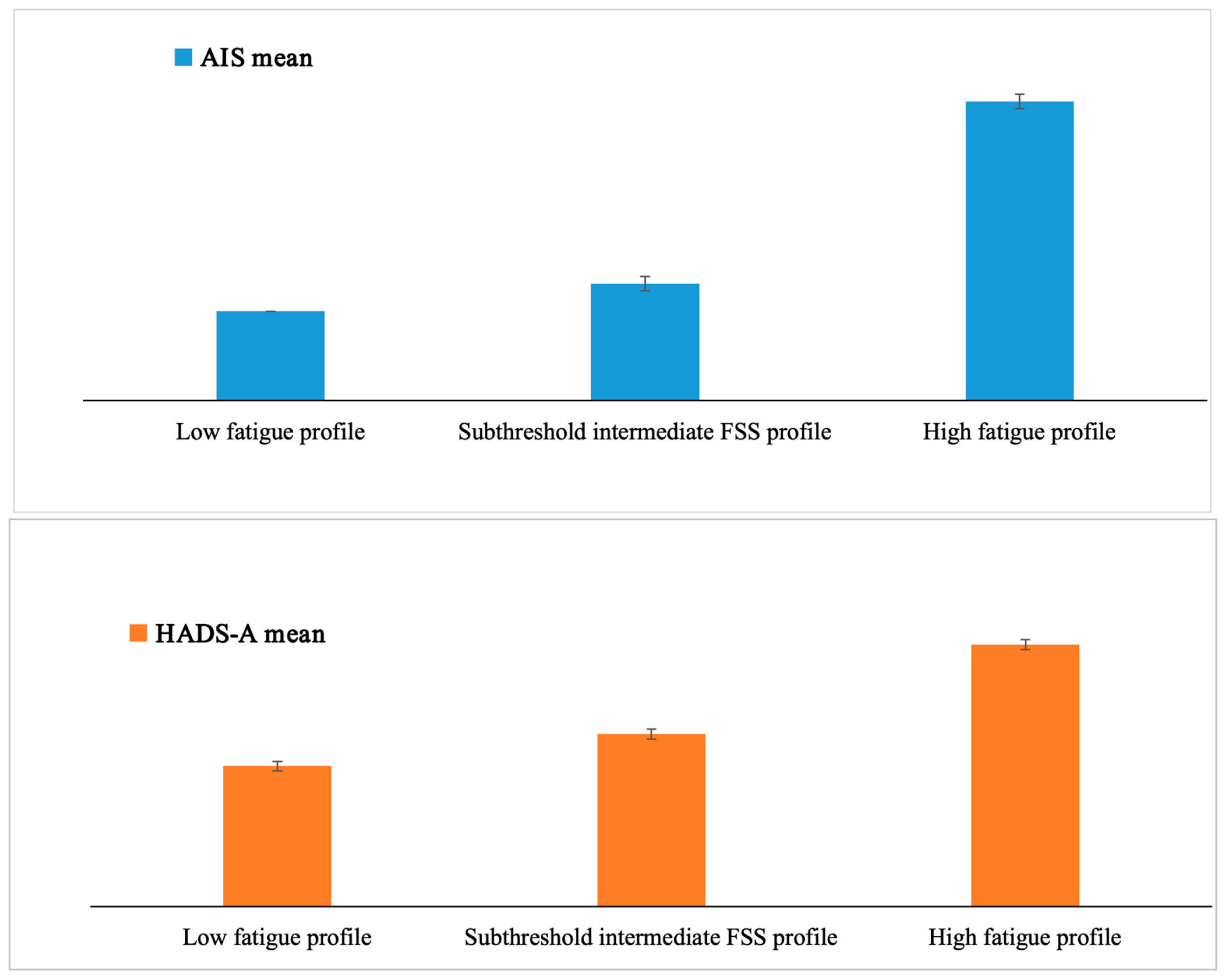

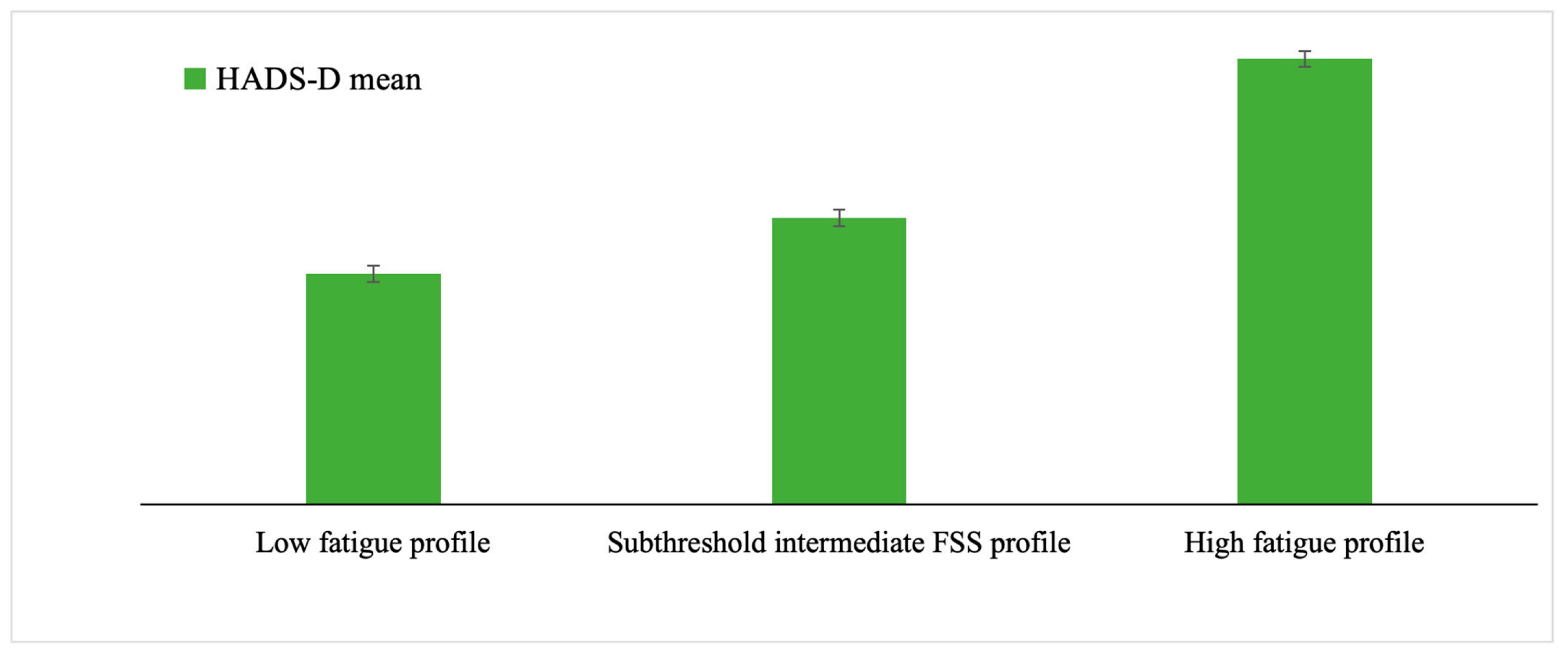
AIS and HADS scores across the three fatigue profiles in patients with first-ever stroke. (AIS = Athens Insomnia Scale; HADS-A = Hospital Anxiety and Depression Scale—Anxiety subscale; HADS-D = Hospital Anxiety and Depression Scale—Depression subscale. Error bars represent standard errors (SE)).

The BCH method was used to compare auxiliary variables across the three latent profiles. Wald chi-square values are shown in Table 3. Significant differences were observed across profiles for anxiety symptoms, depressive symptoms, and sleep quality. Sleep quality differed significantly across profiles (Figure 2). The low FSS profile had lower AIS scores than the subthreshold intermediate FSS profile (χ^2^ = 6.98, *p* = 0.008) and the high FSS profile (χ^2^ = 333.39, *p* < 0.001). The subthreshold intermediate FSS profile also had lower AIS scores than the high FSS profile (χ^2^ = 246.70, *p* < 0.001). Similarly, anxiety and depressive symptoms varied significantly across profiles (Figure 2). The low FSS profile had lower HADS-A and HADS-D scores than the subthreshold intermediate FSS profile (χ^2^ = 20.34, *p* < 0.001; χ^2^ = 20.68, *p* < 0.001, respectively) and the high FSS profile (χ^2^ = 317.37, *p* < 0.001; χ^2^ = 350.71, *p* < 0.001, respectively). The subthreshold intermediate FSS profile also had lower scores than the high FSS profile on both subscales (HADS-A: χ^2^ = 154.44, *p* < 0.001; HADS-D: χ^2^ = 183.42, *p* < 0.001).

Using the established clinical cut-offs, the proportion of patients exceeding the threshold for anxiety symptoms (HADS-A > 7) was 1.9% (3/154) in the low FSS profile, 11.4% (19/166) in the subthreshold intermediate FSS profile, and 67.4% (155/230) in the high FSS profile. For depressive symptoms (HADS-D > 7), the corresponding proportions were 0.6% (1/154), 5.4% (9/166), and 61.3% (141/230), respectively. Regarding sleep disturbance (AIS > 6), the proportions were 3.9% (6/154), 4.8% (8/166), and 57.8% (133/230), respectively. These findings indicate that the high FSS profile had the highest burden of clinically significant anxiety, depressive, and sleep symptoms, while the low FSS profile consistently had the lowest proportions across all three measures (Table 4).

## 4. Discussion

The present study used LPA to characterize the heterogeneity of PSF among patients following an initial stroke event. By identifying distinct subgroups, this research showed significant variations in anxiety, depressive symptoms, and sleep quality across the identified profiles. These findings provide a comprehensive characterization of the co-occurring psychological and physiological factors that capture specific patient phenotypes during the acute or sub-acute recovery phases.

### 4.1. The Heterogeneity of PSF Among Patients with First-Ever Stroke

Latent profile analysis identified three distinct subgroups of PSF, with all fit indices indicating excellent model fit. These findings suggest that PSF in first-ever stroke patients is a markedly heterogeneous phenomenon. Among the participants, 28.0% were classified into the “low FSS profile,” in which the scores on each item were the lowest among the three profiles, reflecting a relatively low level of fatigue-related symptoms. Another 30.2% fell into the “subthreshold intermediate FSS profile,” with item scores lying between those of the other two groups. The remaining 41.8% were assigned to the “high FSS profile,” where the scores on all items were the highest, reflecting that these patients had the most pronounced level of fatigue-related symptoms. These subgroup differences provide further evidence that post-stroke fatigue is a heterogeneous phenomenon. Only the high FSS profile exceeded the clinical FSS cut-off of 4. Our results are consistent with those of Kjeverud [34]; however, they diverge from those of De Doncker [35], who identified four subgroups through cluster analysis. Such discrepancies likely reflect variations in sample characteristics or the specific clustering techniques employed across studies.

Prior research has reported that the prevalence of PSF ranges from 29% to 77% [36]. Consistent with these data, 41.8% of participants met the cutoff for clinical fatigue (high FSS profile), and another 30.2% presented subthreshold intermediate fatigue-related symptoms, indicating that elevated fatigue-related symptoms are prevalent among patients with first-ever stroke. Thus, PSF may represent a common yet frequently under-recognized condition within this clinical population. This condition appears to be associated with extended treatment duration, reduced effectiveness of rehabilitative therapy, and impaired neurological recovery. As a result, patients may face considerable difficulty in re-engaging with normal social, familial, and occupational roles, ultimately affecting their quality of life [37]. Therefore, healthcare professionals may consider prioritizing enhanced clinical guidance and patient education. By improving patient health literacy and implementing targeted, individualized interventions, clinicians may more effectively alleviate fatigue severity and improve long-term outcomes.

### 4.2. Relationship Between PSF Subgroups and Anxiety/Depression as Well as Sleep Levels

Our study showed significant variations in anxiety, depressive symptoms, and sleep quality across the three distinct PSF subgroups. Specifically, scores for anxiety, depressive symptoms, and sleep disturbances increased monotonically with greater PSF severity. Collectively, these findings point to a positive relationship between fatigue levels and the intensity of both psychological distress and sleep disturbances, suggesting that higher fatigue co-occurs with poorer mental health outcomes and reduced sleep quality.

The high FSS profile had significantly higher levels of anxiety and depressive symptoms than the low FSS profile, consistent with previous studies. For instance, a systematic review by Wu et al. [38] reported a moderate positive correlation between PSF severity and both anxiety and depressive symptoms, noting that patients with higher fatigue levels tended to have more severe emotional symptoms. Likewise, a longitudinal cohort study using the Hospital Anxiety and Depression Scale to assess post-stroke anxiety symptoms found that higher fatigue levels were positively correlated with greater anxiety at 1, 6, and 12 months post-stroke (r = 0.50, 0.52, and 0.59, respectively) [39].

This pattern of findings suggests that the high FSS profile carries a particularly heavy psychological burden. As a chronic neurological condition, stroke frequently imposes various forms of suffering on patients, with anxiety and depressive symptoms being especially prominent [40]. From a clinical perspective, anxiety may stem from concerns about the uncertainty of disease prognosis, apprehension about social reintegration, and a sense of helplessness arising from dependence on others for daily living. Depressive symptoms, by contrast, appear more closely tied to changes in social roles, diminished independence, and eroded self-worth. Across systematic reviews, a consistent consensus has emerged that PSF shows a robust association with anxiety and depressive symptoms, with 29% to 34% of patients reporting both conditions [41]. Of note, this prevalence is substantially higher than that observed in the general population—a difference that may reflect the protracted clinical course of stroke and its frequent recurrences, which are known to be associated with heightened helplessness and reduced social engagement in prior studies, potentially contributing to the accumulation of anxiety and depressive symptoms. In addition, severe fatigue may co-occur with persistent weakness and somatic discomfort. This overlapping physical and psychological distress may interfere with patients’ ability to navigate the challenges of the acute stroke phase, and may be associated with anxiety, depressive symptoms, diminished self-esteem, and a weakened sense of self-worth [42].

Consistent with previous work [43], we observed that higher fatigue levels were associated with poorer quality of life. Sustained high-level fatigue tended to coincide with a gradual decline in physical capacity, which may compromise daily activities and hinder fundamental tasks such as walking and eating. As a result, patients may become dependent on others or on assistive devices—a situation that may, in turn, erode their autonomy and undermine self-worth. As physical capacity continues to deteriorate, patients often withdraw from social activities. The resulting prolonged activity restriction and social isolation tend to generate feelings of helplessness and frustration, which may amplify anxiety and depressive symptoms.

The mean AIS score in the high FSS profile was also significantly higher than that in the subthreshold intermediate FSS and low FSS profiles. Moreover, sleep disturbances are common among stroke patients [44]. A cross-sectional study found that one year after stroke, patients’ PSQI scores and daytime sleepiness scores were significantly associated with fatigue levels (r = 0.306 and 0.370, respectively) [45]. Rahamatali et al. [46] also reported a positive relationship between fatigue and sleep disturbances in patients during the chronic phase of stroke. Approximately 50% of stroke survivors report sleep disturbances—a phenomenon that may reflect both brain injury from the stroke itself and environmental factors such as an unfamiliar hospital setting. Together, these elements may compromise sleep quality, which in turn appears linked to impaired physical recovery and fatigue [47,48].

### 4.3. Differences in Demographic Characteristics Across the PSF Subgroups

The present study observed that individuals aged 60 years or older constituted a substantial 60.4% within the high FSS profile, a proportion markedly exceeding that observed in the general population. This observation is consistent with China‘s ongoing demographic shift towards an aging population, as demographic projections indicate that the proportion of geriatric individuals in China is anticipated to surpass 20% by 2033 and reach 35% by 2060 [49]. Additionally, numerous studies have consistently shown that fatigue prevalence and severity tend to increase with advancing age [50,51]. Notably, a study [52] found that post-stroke fatigue can be the only persistent sequela in elderly patients who otherwise show robust physical mobility and psychological recovery.

In descriptive comparisons, other factors—including educational attainment, employment status, monthly income, and residential location—were also observed to be associated with fatigue severity. Specifically, individuals with less than primary school education, unemployment or unstable employment, a monthly income of ≤1000 CNY, and residence in rural areas were more likely to be in the high FSS profile. However, these observations are descriptive and unadjusted; we did not perform multivariable analyses to test independent associations. Therefore, we cannot determine whether these factors independently contribute to fatigue severity or whether their effects are confounded by intercorrelations among socioeconomic variables (e.g., lower education is often associated with lower income and rural residence). Additionally, stroke severity (NIHSS) may confound the relationship between demographic factors and fatigue, as patients with more severe strokes may also be older, have more lesions, and experience greater disability.

Nevertheless, the observed associations may be explained by several interrelated factors. For instance, patients with lower educational attainment often have difficulty understanding complex disease information, show reduced health information-seeking ability, and receive less social support. This situation, combined with limited familial and community support, may be related to treatment adherence and self-management capacity. Furthermore, socioeconomic limitations are often linked to inadequate access to rehabilitation resources (e.g., fewer physical therapy sessions) [53], poorer nutritional status that may influence energy metabolism, and greater financial strain that may be associated with anxiety and depressive symptoms [54]. For rural residents, limited formal employment opportunities, inadequate health insurance, and poor access to medical and specialized healthcare facilities may impede timely and standardized care. Moreover, the dissemination of health-related information remains comparatively restricted in these areas. Taken together, these factors may collectively relate to higher economic pressure and psychological burden, which may be associated with the prevalence and severity of fatigue among stroke patients.

NIHSS scores and the extent of cerebral lesions were associated with fatigue severity. One study that followed 149 stroke patients for at least six months post-onset found that when comparing individuals with NIHSS scores > 3 with those with scores ≤ 3, fatigue prevalence was 87% and 48%, respectively (OR 6.96, 95% CI 1.30–49.25, *p* < 0.05) [15]. Another study also reported an association between fatigue at six months post-stroke and stroke severity [55]. Both studies indicate a positive relationship between stroke severity and fatigue.

Based on these cross-sectional findings, several practice implications emerge, though they require prospective validation. First, routine FSS screening could be integrated into stroke care at admission, during hospitalization, and at discharge, with further HADS and AIS assessment for patients scoring FSS ≥ 4. Second, fatigue assessment may be prioritized during the acute or early sub-acute phase and repeated before discharge to facilitate early identification. Third, a multidisciplinary team could adopt a stratified rehabilitation approach tailored to each fatigue profile. Fourth, nurses could provide frontline interventions, including patient education, energy conservation techniques, fatigue monitoring, and adherence support. Fifth, systematic sleep assessment and management—encompassing sleep hygiene education, environmental optimization, and polysomnography referral when indicated—could be embedded in post-stroke care. Collectively, these strategies offer a framework for stratifying patients by fatigue severity and associated psychological-sleep symptoms, warranting future research to determine their impact on rehabilitation outcomes and quality of life.

Importantly, these recommendations stem from cross-sectional associations and are not directly supported by interventional evidence. Whether this framework improves clinical outcomes remains to be tested in future longitudinal and interventional studies.

### 4.4. Limitations

Several limitations of this study should be acknowledged. First, the single-center design and convenience sampling method may limit the generalizability of the findings. All participants were recruited from one tertiary hospital in northwestern China. Socioeconomic conditions, healthcare access patterns, and cultural factors in this region may differ from those in other areas of China. Additionally, convenience sampling may have introduced selection bias. Therefore, the identified fatigue profiles may not fully represent the stroke population in China or in other cultural contexts. Second, the cross-sectional design precludes any inference about temporal changes in fatigue profiles or their longitudinal associations with psychological and sleep-related variables. Moreover, we cannot determine whether the identified profiles remain stable over time or evolve as patients progress through recovery. Future longitudinal studies are needed to examine the stability and evolution of these fatigue subtypes over time and their relationships with psychological and sleep variables. Third, the use of self-report measures may introduce reporting bias. Furthermore, the reliance on patient-reported data without objective physiological or actigraphic measures of sleep may limit the precision of sleep disturbance assessment. Fourth, the exclusion of patients with severe paralysis may have reduced the representativeness of the sample and may have excluded individuals at higher risk of fatigue. Similarly, the exclusion of patients with recent depressive disorders may have influenced the observed associations between fatigue and psychological symptoms, potentially leading to an underestimation of the relationship between post-stroke fatigue and psychiatric morbidity. Fifth, we did not perform multivariable analyses to test the independent associations between demographic/clinical factors and fatigue profile membership. Therefore, the observed associations should be interpreted as descriptive rather than as evidence of independent effects.

Finally, the lack of longitudinal follow-up prevents any conclusion about the stability of the identified profiles over time. Given these limitations, future multicenter and multiregional studies with larger samples, objective measurements, and longitudinal designs are warranted to validate these findings and improve generalizability.

## 5. Conclusions

The present study identified significant heterogeneity in PSF among patients experiencing their first-ever stroke, and classified patients into three distinct subgroups: low FSS profile, subthreshold intermediate FSS profile, and high FSS profile. BCH method analysis revealed a significant upward trend in both AIS and HADS scores across these three subgroups. Specifically, patients in the high FSS profile had significantly higher AIS and HADS scores than those in the low FSS profile, suggesting an association between fatigue severity, sleep quality, and psychological distress.

## Figures and Tables

**Table 1:** Univariate analysis of patients’ general information and fatigue latent profiles [n (%)].

Variable	Low FSS Profile (154)	Subthreshold Intermediate FSS Profile (166)	High FSS Profile (230)	Test Statistic	*p*
Gender				4.035 ^1^	0.133
Male	112 (72.7)	118 (71.1)	147 (63.9)		
Female	42 (27.3)	48 (28.9)	83 (36.1)		
Residence				7.292 ^1^	0.026
Rural	73 (47.4)	84 (50.6)	139 (60.4)		
Urban	81 (52.6)	82 (49.4)	91 (39.6)		
Age (years)				22.329 ^1^	<0.001
18–45	28 (18.2)	8 (4.8)	15 (6.5)		
45–60	53 (34.4)	64 (38.6)	76 (33.1)		
>60	73 (47.4)	94 (56.6)	139 (60.4)		
Education level				23.076 ^1^	<0.001
Primary school	62 (40.3)	69 (41.6)	113 (49.1)		
Junior high school	41 (26.6)	46 (27.7)	70 (30.5)		
Senior high school or technical secondary school	19 (12.3)	33 (19.9)	33 (14.3)		
College degree or above	32 (20.8)	18 (10.8)	14 (6.1)		
Marital status				10.429 ^1^	0.108
Unmarried	2 (1.3)	4 (2.4)	3 (1.3)		
Married	144 (93.5)	159 (95.8)	212 (92.2)		
Divorced	2 (1.3)	1 (0.6)	0 (0)		
Widowed	6 (3.9)	2 (1.2)	15 (6.5)		
Employment status				22.123 ^1^	<0.001
Employed	69 (44.8)	52 (31.3)	71 (30.9)		
Retired	43 (27.9)	44 (26.5)	43 (18.7)		
Unemployed or waiting for employment	42 (27.3)	70 (42.2)	116 (50.4)		
Monthly income				27.549 ^1^	<0.001
≤1000	2 (1.3)	15 (9.0)	17 (7.4)		
1000–3000	68 (44.2)	77 (46.4)	134 (58.2)		
3000–5000	53 (34.4)	57 (34.4)	60 (26.1)		
>5000	31 (20.1)	17 (10.2)	19 (8.3)		
Number of chronic diseases				3.154 ^1^	0.789
0	39 (25.3)	42 (25.3)	60 (26.1)		
1	73 (47.4)	84 (50.6)	105 (45.6)		
2	33 (21.4)	32 (19.3)	45 (19.6)		
3	9 (5.9)	8 (4.8)	20 (8.7)		
Stroke lesion location				2.934 ^1^	0.817
Left	45 (29.2)	44 (26.5)	66 (28.7)		
Right	42 (27.3)	38 (22.9)	54 (23.5)		
Bilateral	35 (22.7)	51 (30.7)	63 (27.4)		
Other	32 (20.8)	33 (19.9)	47 (20.4)		
Number of lesions				19.356 ^1^	0.08
1	70 (45.5)	57 (34.3)	63 (27.4)		
2	43 (27.9)	55 (33.1)	77 (33.5)		
3	25 (16.2)	33 (19.9)	47 (20.4)		
4	8 (5.2)	11 (6.6)	25 (10.9)		
5	7 (4.6)	6 (3.6)	14 (6.1)		
6	1 (0.6)	4 (2.5)	3 (1.3)		
7	0 (0)	0 (0)	1 (0.4)		
NIHSS score [points, M (P25, P75)]	2.00 (1.00, 3.00)	2.00 (1.00, 3.00)	3.00 (2.00, 7.00)	67.083 ^2^	<0.001
mRS score [points, M (P25, P75)]	1.00 (1.00, 2.00)	1.00 (1.00, 2.00)	1.00 (1.00, 2.00)	3.548 ^2^	0.170
HADS score [points, M (P25, P75)]	9.00 (6.00, 10.00)	10.00 (8.00, 12.00)	16.00 (13.00, 18.00)	259.142 ^2^	<0.001
AIS score [points, M (P25, P75)]	2.00 (0.00, 3.00)	3.00 (2.00, 4.00)	7.00 (5.00, 9.00)	254.405 ^2^	<0.001

Note: Categorical variables were analyzed using the chi-square (χ^2^) test. Non-normally distributed continuous or ordinal variables (NIHSS, mRS, HADS, AIS) were analyzed using the Kruskal–Wallis H test, with the test statistic reported as H. Data for these variables are presented as median (P25, P75). ^1^ χ^2^ value; ^2^ H value.

**Table 2:** Fit indices for latent profile analysis of fatigue in patients with first-ever stroke.

Model	AIC	BIC	aBIC	Entropy	BLRT (*p*-Value)	LMR (*p*-Value)	Category Probability (%)	Average Posterior Probability
1	15,975.57	16,053.15	15,996.01	—	—	—	1.000	—
2	12,754.11	12,874.79	12,785.90	0.97	<0.001	<0.001	0.558/0.442	—
3	12,199.02	12,362.8	12,242.17	0.94	<0.001	<0.001	0.280/0.302/0.418	0.956/0.954/0.995
4	11,976.64	12,183.51	12,031.14	0.91	<0.001	<0.001	0.275/0.111/0.298/0.316	0.963/0.915/0.955/0.957
5	11,945.09	12,195.07	12,010.95	0.86	<0.001	0.389	0.138/0.145/0.291/0.111/0.315	—

**Table 3:** Comparison of auxiliary variables across the three latent profiles.

Variable	Low FSS Profile Mean (SE)	Subthreshold Intermediate FSS Profile Mean (SE)	High FSS Profile Mean (SE)	Overall Test (χ^2^)	Pairwise Comparisons	χ^2^	*p*
AIS	2.018 (0.162)	2.645 (0.161)	6.749 (0.203)	χ^2^ = 372.32	Low vs. Subthreshold Low vs. High Subthreshold vs. High	6.98 333.39 246.70	0.008 <0.001 <0.001
HADS-A	4.198 (0.138)	5.161 (0.152)	7.834 (0.150)	χ^2^ = 336.56	Low vs. Subthreshold Low vs. High Subthreshold vs. High	20.34 317.37 154.44	<0.001 <0.001 <0.001
HADS-D	3.952 (0.141)	4.907 (0.145)	7.630 (0.137)	χ^2^ = 385.36	Low vs. Subthreshold Low vs. High Subthreshold vs. High	20.68 350.71 183.42	<0.001 <0.001 <0.001

**Table 4:** Proportions of patients exceeding clinical cut-offs for anxiety, depressive symptoms, and sleep disturbance across the three latent profiles.

Variable	Low FSS Profile (n = 154)	Subthreshold Intermediate FSS Profile (n = 166)	High FSS Profile (n = 230)
HADS-A > 7, n (%)	3 (1.9%)	19 (11.4%)	155 (67.4%)
HADS-D > 7, n (%)	1 (0.6%)	9 (5.4%)	141 (61.3%)
AIS > 6, n (%)	6 (3.9%)	8 (4.8%)	133 (57.8%)

## Data Availability

The data that support the findings of this study are available on request from the corresponding author. The data are not publicly available due to privacy.

## Abbreviations

The following abbreviations are used in this manuscript:

PSF	Post-stroke fatigue
HRQoL	Health-related quality of life
AIC	Akaike Information Criterion
BIC	Bayesian Information Criterion
aBIC	Sample-size adjusted Bayesian Information Criterion
ADL	Activities of daily living
LPA	Latent profile analysis
LMR	Lo–Mendell–Rubin adjusted likelihood ratio test
BLRT	Bootstrap likelihood ratio test
BCH	Bolck–Croon–Hagenaars method
